# Indentation and Detachment in Adhesive Contacts between Soft Elastomer and Rigid Indenter at Simultaneous Motion in Normal and Tangential Direction: Experiments and Simulations

**DOI:** 10.3390/biomimetics8060477

**Published:** 2023-10-07

**Authors:** Iakov A. Lyashenko, Valentin L. Popov, Vadym Borysiuk

**Affiliations:** 1 Department of System Dynamics and Friction Physics, Institute of Mechanics, Technische Universität Berlin, 10623 Berlin, Germany; v.popov@tu-berlin.de (V.L.P.); v.borysiuk@campus.tu-berlin.de (V.B.); 2 Department of Applied Mathematics and Complex Systems Modeling, Faculty of Electronics and Information Technology, Sumy State University, 40007 Sumy, Ukraine; 3 Department of Nanoelectronics and Surface Modification, Faculty of Electronics and Information Technology, Sumy State University, 40007 Sumy, Ukraine

**Keywords:** adhesion, simultaneous normal and tangential contact, elastomer, friction, adhesive strength, work of adhesion, experiment, simulation

## Abstract

In reported experiments, a steel indenter was pressed into a soft elastomer layer under varying inclination angles and subsequently was detached under various inclination angles too. The processes of indentation and detachment were recorded with a video camera, and the time dependences of the normal and tangential components of the contact force and the contact area, as well as the average contact pressure and average tangential stresses, were measured as functions of the inclination angle. Based on experimental results, a simple theoretical model of the indentation process is proposed, in which tangential and normal contacts are considered independently. Both experimental and theoretical results show that at small indentation angles (when the direction of motion is close to tangential), a mode with elastomer slippage relative to the indenter is observed, which leads to complex dynamic processes—the rearrangement of the contact boundary and the propagation of elastic waves (similar to Schallamach waves). If the angle is close to the normal angle, there is no slipping in the contact plane during the entire indentation (detachment) phase.

## 1. Introduction

Mechanical contacts involving adhesion are very common in biological systems. Adhesive interactions occur both at the microscopic level (the surface adhesion of living cells, viruses and bacteria) and macro level (the ability of certain animals to move on vertical surfaces and ceilings) [1,2,3,4,5]. Different animal species may use different mechanisms of adhesion for their motion abilities. For instance, certain species exhibit attachment to the surface by means of capillarity or by direct adhesive contact or a combination of both mechanisms [6].

Among the most popular examples that illustrate adhesive contacts in nature is the attachment and detachment of the feet of gecko lizards to surfaces that can be smooth or rough. The extraordinary ability of geckos to walk on ceilings is ensured by many keratinous hairs, called setae, on their pads, where each seta is 2–10 μm wide and about 100 μm long. At the submicron level, setae consist of numerous protruding structures known as spatula (≈200 nm wide and long) [6,7]. Thus, in general, the attachment of the gecko foot to the surface can be considered as a large number of the much smaller discrete contacts. Complex structures that consist of setae are also found in other animals. For example, abalones have setae with an average diameter of around 1 μm [8], which is similar to the size of a gecko’s setae.

The strength of the mechanical contact, which must be strong enough to hold the weight of the animal, and their ability to quickly switch between attachment and detachment are also of big interest. It is known that this feature is ensured by different angles at which the animal places and detaches its foot to and from the surface [7,9].

Directly studying the adhesive contacts involving biological objects is a difficult challenge even in laboratory conditions as it typically involves experiments with animals [9]. As was mentioned above, one of the distinct features of the adhesive contacts between the feet of certain animals and a surface is the hierarchical structure of the pads, which firmly covers the surface roughness, providing a larger contact area. Therefore, one possible way to mimic these contacts in laboratory conditions without using animals is to study the indentation of the elastomer substrate with a rigid indenter. The soft surface of the elastomer can also fill the small asperities at the rigid indenter because a soft substrate can be significantly deformed and can fill gaps between asperities, especially in adhesive contacts.

It is worth noting that the problem of the tangential contact and detachment of the soft surface involving adhesion arises not only in the biological environment; it is also an important topic among many scientific groups working in the fields of tribology, contact mechanics, engineering and even modern robotics [10]. Classical adhesion theories such as JKR, DMT and Maugis theory [11,12,13] cannot provide exact general solutions even in the easiest case of pure normal contact, and these theories cannot describe significant differences between the indentation and detachment phases, which are detected in real contacts [14,15]. Much more complicated tangential adhesive contacts are often described within various computational approaches. During recent decades, several techniques for studying the tangential contacts of elastomers involving adhesion that focus on numerical simulations and modeling of the contact phenomena were developed. For example, in [16], the authors use finite element analysis (FEA) to study the adhesion strength of the sliding contacts, taking into account the surface roughness. Moreover, FEA was successfully applied to study adhesion in living cells in [17], where the authors also used atomic force microscopy in their experiments. In [18], the authors developed a model for mixed lubrication that includes adhesion, plastic deformation and surface topography by using the finite element method. Besides FEA, a boundary element method (BEM) is another powerful computing tool that is frequently used to study mechanical contacts. Recently, it was applied to study the effect of adhesion and surface roughness on friction hysteresis [19]. Computational studies of friction and adhesion between polymers at the nanoscale are typically performed within the framework of molecular dynamics (MD) simulations where special approaches for tangential contacts were also developed (see, for example, [20,21]). As another illustrative example of the modeling of tangential adhesive contact, we can refer to the model based on traction–separation laws developed in [22], the n-point asperity model proposed in [23], the fracture mechanics model [24] and many others (see, for example, [25,26]).

Besides theoretical studies, tangential contacts are also investigated experimentally. For this purpose, special laboratory facilities were developed. A famous example of experimental techniques for simultaneous measurements of both the normal and tangential forces between soft surfaces is the special version of the classical surface force apparatus [27]. In a more recent study, a rotary shear apparatus was used to study the tangential adhesion strength between clay and steel in [28]. Important examples of laboratory equipment developed to measure tangential adhesion are special tribometers (see, for instance, [29,30]). There are many studies in which the authors use relatively simple self-developed experimental setups. As a rule, that is enough for the investigation of adhesive contacts in biological macroscopic organisms (animals) [8]. But, despite the large number of experimental and theoretical works, adhesive tangential contact is still the object of hot discussions in various scientific groups. This is due to complex processes during contact area restructuring at tangential motion (attachment of new contact areas, peculiarities of detachment, adhesive hysteresis at changing of motion direction, partially sliding, elastic waves propagation, pores formation during motion, etc.) and importance for practical applications.

Here, we present a series of experiments and mathematical simulations concerning both indentation of the rigid indenter into soft elastomer and also withdrawal of the indenter from elastomer simultaneously in normal and tangential directions. Also, we propose a simple model for normal and tangential contact within the method of dimensionality reduction (MDR) for adhesive contact [31]. We believe that the performed study can bring new insights into understanding of formation and detachment of the biomechanical contacts.

## 2. Experimental Set-Up

**Figure 1:**
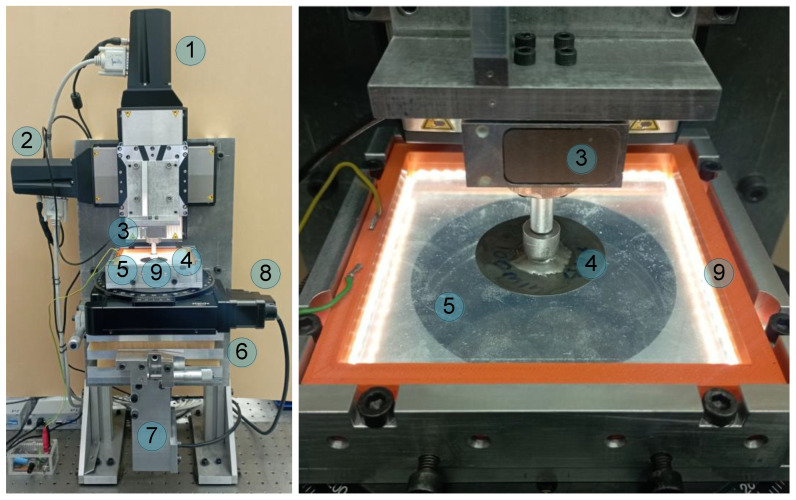
(**left panel**) Photo of the whole experimental facility; (**right panel**) enlarged photo of the contact area between a spherical indenter 4 and elastomer 5 with all-sides LED lighting 9.

To perform described below experiments a special type of laboratory equipment, that is shown in Figure 1 was designed and assembled.

The left panel of the figure shows a general view of the designed facility, whereas the right panel demonstrates only the area of the contact. Both photos of the facility depict the main parts of the experimental device. The spherical indenter made of steel (denoted as (4) in the figure), mounted onto three-axial force sensor ME K3D40 (denoted as (3)). During the experiment, the force sensor measures all three components of normal force. Electric signal from the sensor (3) is amplified by 4-channel amplifier GSV-1A4 SubD37/2 with three out of four active channels for each component of the force. Outputs of the amplifier paired with desktop PC through the 16-bit analogue to digital converter NI USB-6211. Indenter is capable of moving in both normal and tangential directions as it is driven by two high-precise motors PI L-511.24AD00 (1) and (2), respectively, that are governed by USB controllers PI C-863. For more precise positioning, the mentioned motors are equipped with a feedback mechanism that automatically measures and corrects (if needed) the coordinate after movement. With the presence of the feedback, the accuracy of positioning may reach values up to ±0.2 μm. Another important option of the drives that are used in the facility is a lack of hysteresis of coordinate after changing the direction of motion. Such a type of hysteresis almost always exists in devices without feedback due to the inevitable backlash in the mechanical converters of rotary motion into translational motion, even in devices with more accurate and expensive ball-screw gears.

Sheet of transparent rubber TARNAC CRG N3005 with thickness *h* = 5 mm was used as an elastomer substrate (5), and almost perfect transparence of this rubber ensures the possibility of direct observation of the contact area. This type of rubber is characterized by elastic modulus *E* ≈ 0.324 MPa and Poisson’s ratio *ν* ≈ 0.48, which were estimated in [32] by generalizing a large amount of experimental data. Elastic modulus of the indenter material (steel) is 5 orders of magnitude higher and equals *E* ≈ 2 × 10^5^ MPa, therefore in experiments the indenter can be considered as absolutely rigid and deformations only occur inside the rubber substrate.

Observation of the contact area in experiments is performed from the bottom part of the device through the rubber sheet by digital camera Ximea 2.2MP MQ022CG-CM with FUJINON HF16SA-1, 2/3” lens. In Figure 1 the position of the camera is denoted by (7), whereas the camera itself is closed from the observer by an aluminum plate. Tilt mechanism (6) can be used to manually change the position of the elastomer in the horizontal plane, which is critically important for experiments with tangential motion. Motorized rotation stage 8MR190-90-4247-MEn1 (8), governed by 8SMC5-USB-B8-1 USB-controller, is typically used for the rotation of elastomer; however, in the described experiments, it is idle. The facility shown in Figure 1 was described in detail in our recent work [33], and the provided therein “Supplementary Video” shows the performance of the device in real time.

**Figure 2:**
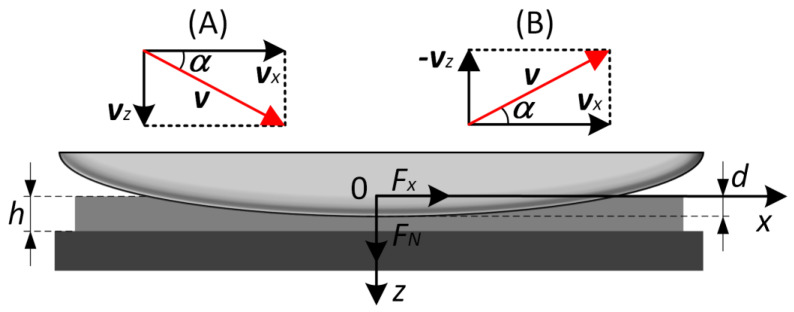
Schematics of two experiments: (**A**) immersion of the indenter into elastomer, and its withdrawal (**B**). In both experiments indenter moves with velocity *v* that has normal *v_z_* and tangential *v_x_* components. Figure shows the configuration with thickness of an elastomer *h*, indentation depth *d*, normal and tangential components of contact force *F_N_* and *F_x_*, and indentation angle *α*.

Here, we describe a series of experiments performed according to scenarios (A) and (B), which are shown in Figure 2. In both cases, in the first phase of experiment during the movement of indenter with radius *R* in normal direction, the point of the first contact between indenter and rubber was detected, and a related normal coordinate was considered as zero indentation depth *d*_0_ = 0 mm. Then, in scenario (A), indenter was immersed into the depth *d*_max_ = 0.3 mm at an angle *α* to the elastomer surface (see Figure 2). Movement of the indenter at a certain angle was performed by its simultaneous displacement, driven by the motors (1) and (2) (see Figure 1) with different velocities *v_z_* and *v_x_*. Velocities *v_z_* and *v_x_* were chosen in such a way that the absolute value of the resulting velocity in each case was the same $v=\sqrt{v_{z}^{2}+v_{x}^{2}}=1$ μm/s (see Figure 2). Thus, at a given indentation angle *α* absolute values of velocities can be determined as *v_x_* = *v* cos*α* and *v_z_* = *v* sin*α*. Note that at such a small velocity of indenter motion, the contact can be considered as a quasi-static (viscoelastic effects do not appear or are very small) as it was experimentally shown in our previous work [34]. It means that the obtained experimental data can be described by classical theories of adhesion of elastic bodies as JKR-type theories.

In scenario (B), the indenter was initially immersed into the elastomer to the depth *d*_max_ = 0.3 mm at motion in a normal direction, and then it was withdrawn from the substrate at an angle *α* to its surface up to full detachment of the contact. Vectors of velocities for both scenarios (A) and (B) are shown in the Figure 2. Several series of experiments were performed with two different indenters of radius *R* = 30 mm and *R* = 100 mm and at angles *α* = 10°, 20°, 30°, 40°, 50°, 60°, 70°, 80°, 90° (angle *α* = 90° related to normal contact). Separately, experiments with pure tangential movement of an indenter were performed (at *α* = 0°), where prior to tangential shift indenter was immersed into elastomer to the depth *d*_max_ = 0.3 mm. In this case, the conditions of the experiments differ from both scenarios (A) and (B).

## 3. Experimental Results

Movement of the indenter at an angle *α* to the elastomer surface includes both normal and tangential shift. Therefore, prior to analyses of the data obtained from indentation at an angle, it is necessary to have an insight of what is happening in simpler cases of pure normal and tangential contacts. In several of our previous works, various aspects concerning normal [32,33,34,35,36] and tangential [33,37,38] contact were considered. However, due to the complexity of the considered phenomena, here we will not only cite our previous works, but additionally will conduct experiments on both normal indentation and tangential shear (the descriptions of the experiments are given in the next two subsections). It is worth noting that these experiments were performed with the same elastomer and indenter, as the experiments with the indentation at an angle *α*. This will help to obtain additional information about the studying system.

### 3.1. Normal Contact

**Figure 3:**
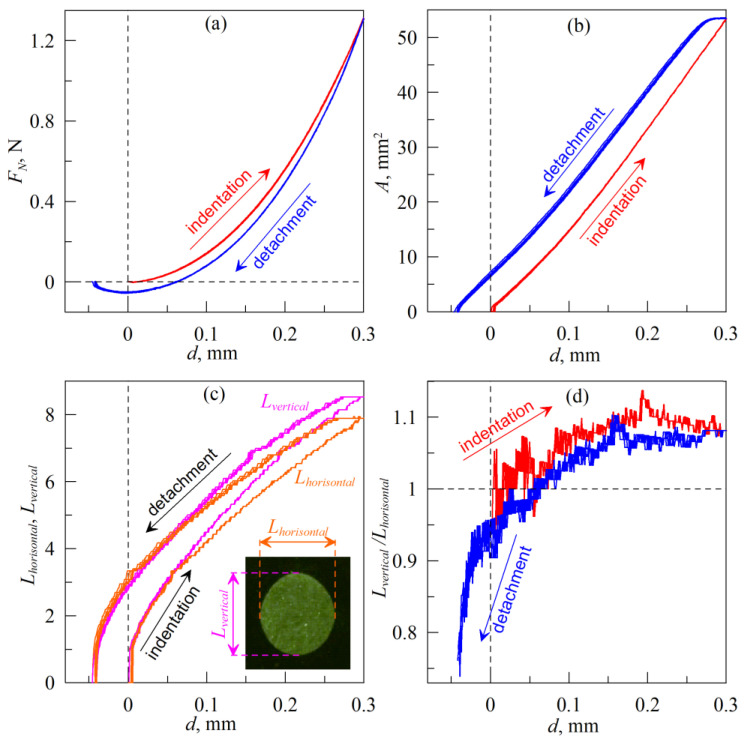
Dependencies of normal force *F_N_* (**a**), contact area *A* (**b**), size of the contact in vertical *L_vertical_* and horizontal *L_horisontal_* directions (**c**) and ratio *L_vertical_*/*L_horisontal_* (**d**) on indentation depth *d*. Radius of an indenter *R* = 30 mm, elastomer (CRG N3005) thickness *h* = 5 mm. Supplementary Video S1 is also available.

Figure 3 shows results of experiments where indenter was moved at an angle *α* = 90° to elastomer plane (see schematics in Figure 2). As it was mentioned above, this situation relates to the indentation in pure normal direction. The figure shows five full indentation/detachment cycles. Overlapping of the curves, corresponding to different cycles, indicates good reproducibility of the experiments.

As it was observed in various experiments on different scales [32,33,34,35,36,37,38,39,40], in the indentation phase, adhesion is neglectable and contact can be considered as non-adhesive. However, in the detachment phase, adhesion significantly affects the properties of the contact, which results in an additional force (*F_N_* < 0 N at *d* < 0 mm) that must be applied for complete detachment of the indenter. Mentioned peculiarities lead to adhesive hysteresis of the second art in the dependencies of the force *F_N_*(*d*) and the contact area *A*(*d*), which is clearly visible in the Figure 3a,b. Reasons which lead to hysteresis are not completely established yet, and in the literature it is explained by the influence of humidity, viscoelasticity or roughness of the contacting surfaces [39]. The presence of the hysteresis causes additional dissipation of the mechanical energy in oscillating adhesive contacts [35]. In a situation where contacting surfaces have a non-uniform distribution of surface energy (which determines the specific work of adhesion), such hysteresis arises naturally within the framework of classical adhesion theories, like JKR [35]. If two hard contacting surfaces have surface energy densities *γ*_1_ and *γ*_2_, and *γ*_12_ is an interfacial energy density for interface between these surfaces in the contact, the specific work of adhesion can be expressed as [41]

(1) $$\Delta \gamma =\gamma_{1}+\gamma_{2}-\gamma_{12}.$$

In the case if Δ*γ* > 0, the contacted surfaces adhere to each other. A simple phenomenological approach to the description of adhesive hysteresis of the second art can be to introduce two effective values of the specific work of adhesion at the stages of indentation Δ*γ_eff_*_,0_ and detachment Δ*γ_eff_*_,1_, where Δ*γ_eff_*_,1_ > Δ*γ_eff_*_,0_. Such a simple assumption makes it possible to simulate the normal adhesive contact with a sufficient accuracy of reproducibility of experimental results [34,36,40]; however, it does not explain the cause of the hysteresis.

It is worth noting that in a real experiment the shape of the indenter is not absolutely spherical and the rubber surface is also not ideally smooth. Therefore, the contact area will deviate from the expected round shape. In this case, one parameter (contact radius) used for description of the contact geometry becomes insufficient. In this regard, in all experiments described below, additional dependencies of the “width” in horizontal *L_horisontal_* and vertical *L_vertical_* directions are introduced (see Figure 3c). Here and below mutually perpendicular “horizontal” *L_horisontal_* and “vertical” *L_vertical_* directions are chosen from the location of the camera in the device shown in Figure 1. For experiments with indentation in the normal direction, these are arbitrary directions that have no geometric meaning. However, if there is tangential movement, the direction *L_horisontal_* coincides with the direction of the tangential shift of the indenter. At the same time, *L_vertical_* will automatically coincide with the direction perpendicular to the direction of the tangential shift. In the case of a circular contact patch, its “width” is the same in both directions and reduced to the diameter of the contact.

### 3.2. Tangential Contact

**Figure 4:**
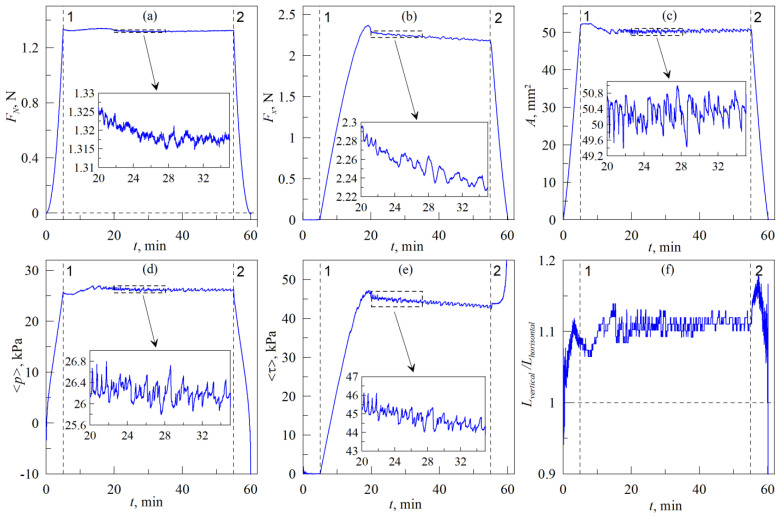
Time dependencies of the normal *F_N_* (**a**) and tangential *F_x_* (**b**) forces, contact area *A* (**c**), average contact pressure <*p*> = *F_N_*/*A* (**d**), averaged tangential stresses <*τ*> = *F_x_*/*A* (**e**) and ratio *L_vertical_*/*L_horisontal_* (**f**). Radius of the indenter *R* = 30 mm, elastomer thickness (CRG N3005) *h* = 5 mm, indentation depth during tangential shift *d*_max_ = 0.3 mm, velocity of the indenter motion *v* = 1 μm/s. Supplementary Video S2 is also available.

Figure 4 shows results of an experiment in which the indenter was first immersed in the normal direction into the rubber to a depth *d*_max_ = 0.3 mm, and then shifted in a tangential direction at a fixed depth *d*_max_. After shifting to a distance Δ*x* = 3 mm, the indenter was withdrawn from the elastomer until the moment of complete detachment of the contact. The figure shows time dependencies of the normal force *F_N_*, tangential force *F_x_*, contact area *A*, average contact pressure <*p*> = *F_N_*/*A*, averaged tangential stresses <*τ*> = *F_x_*/*A*, and ratio *L_vertical_*/*L_horisontal_*. Two vertical dashed lines 1 and 2 are present in all panels of the figure, where up to line 1, indentation in the normal direction is performed (in this case, the tangential force *F_x_* and corresponding tangential stresses are equal to zero). Between lines 1 and 2, the indenter is shifted in tangential direction at fixed indentation depth *d*_max_, and after line 2, the indenter is pulled off the elastomer until the detachment. 

During the phase of experiment until line 1, the dependencies in Figure 4 relate to normal indentation, therefore they have the same features as the data in Figure 3. Curves plotted in Figure 3 relate to the dependencies on the indentation depth *d*, whereas Figure 4 shows time dependencies of the main measured quantities in the experiment. Nevertheless, indentation is performed with constant velocity, therefore both figures have the same peculiarities, such as a linear increase in the size of contact area *A* in Figure 3 and Figure 4 with the growth of indentation depth *d* and time *t*.

A tangential shift of the indenter begins over the line 1 in panels of Figure 4, and after that, the tangential force *F_x_* first increases to a certain maximum value, then decreases. After this decrease in the *F_x_*, the stationary sliding mode begins. In all panels of the figure, except panel (f), immediately after the establishment of the stationary sliding mode, enlarged parts of the curves (that are bounded by dashed rectangles in the main figures) are shown for better clearance. In the enlarged regions, it is easier to trace the relationships between the quantities that are measured in the experiment. For example, in the insets to the panels of the figure, it is clearly visible that all measured quantities change nonmonotonically. This is due to the fact that during the sliding, complex processes of contact propagation occur in the system. These processes are associated with the abrupt appearance of new contact areas between the rubber and the indenter at the leading edge of the contact, with more monotonous-like separation of the rubber at the trailing edge, and also with the propagation of elastic waves in the contact area. These waves are known as “Schallamach waves” [42,43,44,45,46] and it is one of the least understood effects in adhesive contacts. Each subsequent propagation of an elastic wave leads to a decrease in the friction force and tangential stresses, which increase again with further shear. In our previous works [33,37,38], these processes are described in detail, especially in the recent work [38], where we discuss the inhomogeneous nature of slip phase during tangential shear of the indenter. Moreover, in Supplementary Video S2, features of contact propagation during tangential motion are clearly visible.

One particular feature, which can be observed in Figure 4, needs additional explanation. As it follows from the figure, in the mode of stationary sliding, normal force *F_N_* and contact area *A* fluctuate around certain average magnitudes, without any radical changes (either decrease or growth) in time. This means that during tangential movement, the indentation depth *d* = 0.3 mm remains constant, i.e., there are no explicit irregularities on the surface of this particular rubber substrate in the direction of indenter movement. However, despite constant values of the force *F_N_* and area *A*, tangential force *F_x_* decreases in time (see Figure 4b), together with tangential stresses <*τ>* = *F_x_*/*A*. In the considered case of adhesive contact in the established stationary sliding mode, the friction force, regardless of the indentation depth *d*_max_, is determined by expression (without considering friction at the contact boundary [37]):

(2) $$F_{x}\approx \tau_{0}A,$$

where stationary tangential stresses *τ*_0_ depend on adhesive forces between contacting surfaces. In this case, the coefficient of friction *μ* loses its physical meaning as it becomes dependent on indentation depth *d*_max_ [38]. According to (2), friction force *F_x_* is proportional to the contact area *A*, which has almost constant value <*A*> ≈ 50 mm^2^ in stationary mode (see Figure 4c). Therefore, the observed decrease in the friction force is possible only due to the reduction of the stationary stresses *τ*_0_, which can be observed in the Figure 4e. The decreasing of stresses occurs due to the degradation of the adhesive properties of the contact, caused by contamination and oxidation of the indenter surface during the tangential shift. In this case, the specific work of adhesion decreases, which leads to a decrease in the adhesive strength in normal contact (by adhesive strength here we assume the normal force that must be applied to completely detach the indenter from the rubber) [36]. In the case of tangential contact, as it can be seen, the degradation of adhesive properties leads to a decrease in the stationary value of tangential stresses *τ*_0_, and, as a result, to a decreasing of friction force *F_x_*.

Decrease in the adhesive strength of the contact makes comparative analysis of experimental data more difficult, since each individual experiment will have its own value of the specific work of adhesion, on which all other characteristics of the contact such as its radius, contact forces, etc., depend. In further analysis of the experimental data concerning indentation at different angles *α*, we will neglect the changes in the adhesive strength of the contact. In the case considered below, such constraint is relevant, since a change in the angle *α* at which the indentation is performed has a much stronger effect on the contact properties than the observed change in the specific work of adhesion due to degradation of the contact properties.

### 3.3. Immersion of the Indenter at an Angle to the Surface, Scenario (A)

**Figure 5:**
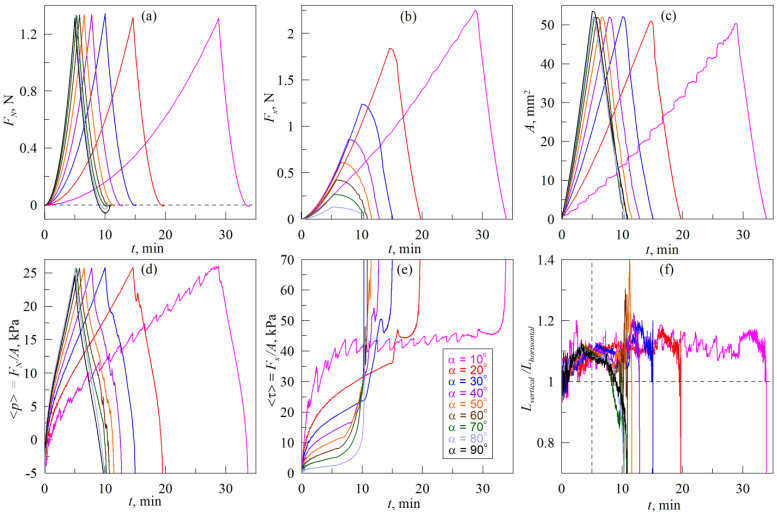
Time dependencies of the normal *F_N_* (**a**) and tangential *F_x_* (**b**) forces, contact area *A* (**c**), average contact pressure <*p*> (**d**), tangential stresses <*τ*> (**e**) and ratio *L_vertical_*/*L_horisontal_* (**f**). Radius of the indenter *R* = 30 mm, elastomer thickness (CRG N3005) *h* = 5 mm, maximal indentation depth *d*_max_ = 0.3 mm, in the experiment according to scenario (A) (see Figure 2). Dependencies corresponding to the experiment with the same angle *α* (from 10° to 90°), plotted in different panels of the figure in the same color. Supplementary Video S3 is available.

Figure 5 shows results of experiments on indentation of the metallic spherical indenter with radius *R* = 30 mm into elastomer sheet TARNAC CRG N3005 with thickness *h* = 5 mm, that was performed according to scenario *A* (see Figure 2 and related description). The figure shows time dependencies of the normal *F_N_* (panel a) and tangential *F_x_* (panel b) components of the contact force, contact area *A* (panel c), average normal pressures <*p*> = *F_N_*/*A* (panel d), average tangential stresses <*τ*> = *F_x_*/*A* (panel e) and ratios *L_vertical_*/*L_horisontal_* (panel f). The group of curves, corresponding to experiments on indentation at angles *α* from 10° to 90° with 10° increments, are plotted. Dependencies corresponding to the same experiment (the same angle *α*), plotted in different panels of the figure in the same color. Moreover, each panel contains nine curves, except for dependencies of the tangential force *F_x_*(*t*) and tangential stresses <*τ>*(*t*) (panels (b) and (e)), where the results of the experiments with *α* = 90° are not shown, because under pure normal load the tangential force and related stresses *τ* do not occur.

In all experiments, presented in Figure 5, after the appearance of the first contact point between the indenter and the rubber, the indenter was pressed into the elastomer at an angle *α* to its surface until reaching the indentation depth *d*_max_ = 0.3 mm. As soon as the depth *d*_max_ was reached, the indenter was withdrawn from the elastomer in a normal direction until the complete detachment of the contact. As it follows from the figure, during the indentation phase, magnitudes of normal *F_N_* and tangential *F_x_* forces as well as contact area *A* and contact pressure <*p*> are increasing. As indentation always continues up to the depth *d* = *d*_max_ = 0.3 mm, *F_N_*, *A* and <*p*> grow to approximately the same maximum values regardless the magnitude of an angle *α*. This happens because the quantities *F_N_*, *A* and <*p*> characterize a normal contact, which is mainly determined by the indentation depth *d*_max_. The main difference between curves *F_N_*(*t*), *A*(*t*) and <*p*>(*t*), corresponding to different angles *α*, consists of different rates of growth, since with an increasing of the angle *α* indenter reaches the maximal depth *d*_max_ faster.

After reaching the maximum values, *F_N_*, *A* and <*p*> begin to decrease, as the phase of indentation at an angle is replaced by the phase of the detachment, where indenter moves in the pure normal direction (detachment in all cases occurs at *α* = 90°).

Let us note some peculiarities of contact propagation during indentation at different angles. As it was observed in previous works, stationary sliding mode is established in the studied system during pure tangential motion. In stationary mode, regardless of the value of the indentation depth *d*, tangential stresses *τ*_0_ = *F_x_*/*A* occur over all contact area of magnitude from 30 to 50 kPa in different experiments (see Figure 4e, and also experimental data reported in [37,38]). Magnitudes of *τ*_0_ vary in different experiments as *τ*_0_ strongly depends on the current state of the contacting surfaces, namely, on the presence of chemical and physical contaminations, roughness, etc. As it follows from the Figure 5, only in the case of indentation at a minimum angle *α* = 10° the indenter is shifted in the tangential direction on the distance enough for tangential stresses *τ* to reach the critical value *τ*_0_ ≈ 40 kPa, above which tangential slip begins. After the stresses reach the value *τ*_0_, it does not increase with further tangential shift, although the friction force *F_x_* and contact area *A* continue to increase. These parameters grow due to the fact that when indenting at an angle *α* to the surface, shift in tangential and normal directions is performed simultaneously, and, due to the increasing of the indentation depth *d*, the contact area *A* growth together with friction force *F_x_* ≈ *τ*_0_ *A* (2).

In the stationary sliding mode, contact propagation has certain features that can be traced on the dependencies *A*(*t*), <*p*>(*t*) and <*τ*>(*t*) in Figure 5. To explain these features, let us return to the case of solely tangential shear, which is shown in Figure 4. Here, during the contact propagation, smooth detachment of the rubber from the indenter occurs on the back front, so the boundary of a contact on the back front practically does not change its shape. At the leading age of the contact, new areas of rubber are involved in the contact with the indenter’s surface in an abrupt manner (see Supplementary Video S2), therefore contact area *A*(*t*) also increases abruptly. Since the new parts of the rubber that have just come into contact are not loaded in the tangential direction, they do not contribute to the friction force *F_x_* immediately after contact. Therefore, such an increase in the contact area *A* without corresponding growth of the friction force *F_x_* leads to a sharp decrease in the average tangential stresses <*τ*> = *F_x_*/*A*. With further shifting, the tangential stresses <*τ*> increase again due to the loading of the “new” areas of the rubber, at the same time the contact area *A* monotonically decreases. The described contact propagation process is repeated cyclically, which can be seen in the dependencies plotted in Figure 4 and in Supplementary Video S2 (for more details, please see related description in [38]).

In experiments on indentation at an angle *α* to the surface of the elastomer, a mode similar to the tangential shift is established in the system at small angles *α*. In this mode, there is also a jump-like mechanism of contact propagation, so dependence *A*(*t*) in Figure 5c at *α* = 10° exhibits regions with sharp growth of the area *A*. However, in contrast to a pure tangential shift, new contact areas appear in an abrupt manner not only at the leading edge of the contact, but along the entire contact boundary, which is clearly visible in Supplementary Video S3. The reason for such behavior is the fact that along with the tangential movement, there is a constant increase in the indentation depth *d*. As in the case of a pure tangential shift, immediately after the appearance of “fresh” areas of contact, they appear to be unloaded in the tangential direction, which is verified by the absence of abrupt-like changes in the tangential force *F_x_* after the attachment of new areas of rubber (see Figure 5b at *α* = 10°). As it follows from Figure 5, abrupt growth of the contact area *A* does not lead to sharp changes in the normal force *F_N_* also. Therefore, after the following act of contact propagation, both calculated values of the averaged tangential stresses <*τ*> = *F_x_*/*A* and contact pressure <*p*> = *F_N_*/*A* decrease. For an adequate description of the evolution of these parameters, it is necessary to take into account the spatial distribution of stresses, as it was discussed in our previous work [38].

Above we described the case of indentation at an angle *α* = 10°, which is close to tangential sliding. In this case, the tangential stresses reach a critical value *τ*_0_ ≈ 40 kPa during the tangential shift, after which the elastomer slips over the surface of the indenter. In all other experiments with *α* > 10°, as it follows from the Figure 5e, tangential stresses <*τ*> did not reach a critical value throughout the entire indentation up to the critical indentation depth *d* = *d*_max_. Therefore, in these experiments, global slippage as well as abrupt-like expansion of the contact area were not observed, during indentation. As it follows from the data plotted in Figure 5 and from the Supplementary Video S3, at *α* > 10° all main parameters, such as both components of contact forces *F_x_* and *F_N_*, contact area *A*, as well as contact pressure <*p*> and tangential stresses <*τ*> increased smoothly and monotonously during the whole indentation phase.

### 3.4. Pull-Off of the Indenter at an Angle to the Surface, Scenario (B)

**Figure 6:**
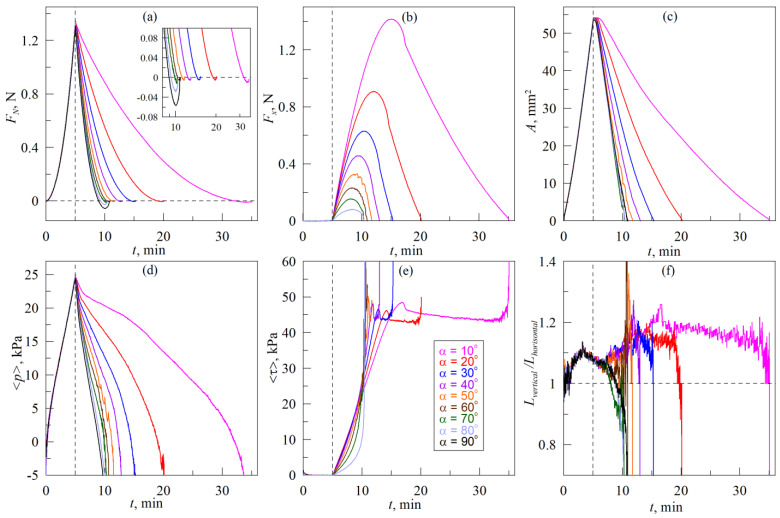
Time dependencies of the normal *F_N_* (**a**) and tangential *F_x_* (**b**) forces, contact area *A* (**c**), average contact pressure <*p*> (**d**), tangential stresses <*τ>* (**e**) and ratio *L_vertical_*/*L_horisontal_* (**f**). Radius of the indenter *R* = 30 mm, elastomer thickness (CRG N3005) *h* = 5 mm, maximal indentation depth *d*_max_ = 0.3 mm, in the experiment according to scenario (B) (see Figure 2). Dependencies corresponding to the experiment with the same angle *α* (from 10° to 90°), plotted in different panels of the figure in the same color. Supplementary Video S4 is also available.

In the experiments, the results of which are shown in Figure 6, the indenter was first immersed into the elastomer to a depth *d*_max_ = 0.3 mm (moved in the normal direction) and then was withdrawn from the elastomer at an angle *α* to its surface until the complete detachment.

Here, the case with *α* = 90°, as in previous Figure 5, relates to only normal indentation. Therefore, analogously to Figure 5, in the panels (b) and (e) of Figure 6 there are no dependencies related to the case with *α* = 90°.

Vertical dashed lines in all panels of Figure 6 show the point of time when indentation in the normal direction ends and the indenter is pulled off of the elastomer when it moves at an angle *α* to its surface until the complete detachment of the contact. It is worth noting that all dependencies *F_x_*(*t*), plotted in Figure 6b, have maximums. Two main factors affect the friction force *F_x_* during the pull-off of the indenter. First, friction force *F_x_* growth due to the increase in the tangential stresses *τ*, as the tangential movement is performed. The second factor that causes the reduction of the tangential force is the decreasing of the contact area *A* due to the detachment of the indenter in normal direction. Moreover, stresses *τ*, and contact area *A* equally affect the friction force, as it is defined by the expression *F_x_* ≈ <*τ*>*A* (2). Based on the fact that in all cases the force *F_x_* first increases and then decreases, we can conclude that at the beginning stresses *τ* grow faster than area *A* decreases. However, with further movement of the indenter, at certain moment the stresses reach a critical value *τ*_0_ ≈ 45 kPa, after which slip occurs in the system, and the shear stresses no longer increase (see Figure 6e). However, in the sliding mode, the contact area *A* continues to decrease due to the pull-off of the indenter in the normal direction, which leads to the reduction of tangential force *F_x_*.

The behavior of the system in the case shown in Figure 6, has a significant difference comparing to the series of experiments described above and presented in Figure 5. In experiment, shown in Figure 6, movement in the tangential direction begins only after the indenter reaches its maximum indentation depth. The contact area in this case is maximal. Therefore, the subsequent tangential movement loads the entire contact at once, while simultaneously moving the indenter in the normal direction. In this case, there is no contact propagation when attaching “new” regions that are non-loaded in the tangential direction, which were observed during indentation according to the scenario (A) (see Figure 5). Dependencies shown in Figure 6, as well as in Supplementary Video S4, show a smooth decrease in the contact area regardless the angle *α*, at which the indenter is pulled off. Therefore, in this case, we can assume that during the entire process of withdrawing of the indenter, new contact regions between the rubber and the indenter are not formed, and only the destruction of the existing contact at its boundary occurs. Such an assumption significantly simplifies the understanding of the processes occurring in the system. If there is only destruction of the contact without its propagation and corresponding rearrangement of the contact boundary, a simpler numerical model can be used for the description, such as the method of dimensionality reduction (MDR) or method of the boundary elements (BEM). Note that these methods give the exact solution of the contact problem for normal contact with adhesive interaction of a JKR type [31,32].

Let us note one important feature. The assumption that during the withdrawing of the indenter there is no contact propagation, but only its destruction, will fail in two cases. The first case is when indenter is pulled off at a very small angle *α*, as at small *α*, contact approaches a completely tangential motion mode, in which the rubber slips and contact inevitably propagates during movement (see Figure 4). The second case is weak adhesive interaction between contacting bodies. Such contacts are characterized by small magnitudes of critical stresses *τ*_0_, at which slips that are always associated with the spread of the contact in the tangential direction, occur. Therefore, at weak adhesion, when moving at an angle, after a relatively small shift of the indenter, the sliding mode will occur, and the accurate description of this mode requires a serious modification of existing models (see, for example, [38]).

## 4. Simulation of the Indentation/Detachment Process

### 4.1. Formalism of the Model

**Figure 7:**
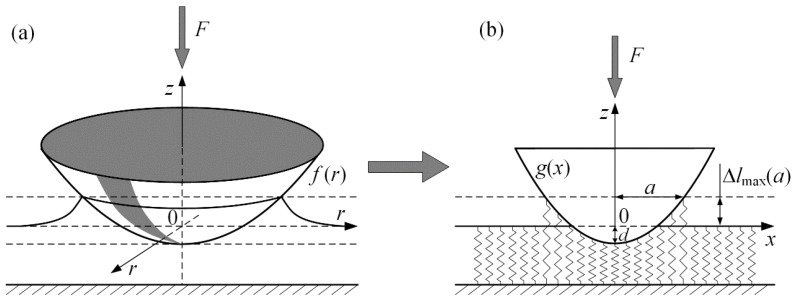
Schematics of the method of dimensionality reduction (MDR) for normal adhesive contact—initial three-dimensional profile (**a**) and corresponding one-dimensional configuration (**b**).

In this section, we describe the simulation of the indentation at an angle in an adhesive contact that is developed from experimental data provided in the previous sections of the article. Simulation setup is based on the method of dimensionality reduction (MDR [31]), the schematics of which for normal adhesive contact are presented in Figure 7.

Within MDR for an axially symmetrical indenter with three-dimensional profile *f*(*r*) an equivalent one-dimensional profile *g*(*x*) must be found according to Abel transform:

(3) $$g(x)=|x|\int_{0}^{\left|x\right|}\frac{f^{\prime }(r)}{\sqrt{x^{2}-r^{2}}}dr.$$

In our experiment, we use a spherical indenter, which, at small indentation depths *d*, can be replaced by a paraboloid *f* (*r*) = *r*^2^/(2*R*) with sufficient accuracy, thus, according to the procedure (3) we obtain:

(4) $$g(x)=\frac{x^{2}}{R}.$$

Then, the elastic half-space is replaced by an array of non-interacting springs (see Figure 7b), each of them has both normal Δ*k_z_* and tangential Δ*k_x_* stiffness:

(5) $$\Delta k_{z}=E^{*}\Delta x,\;\Delta k_{x}=G^{*}\Delta x,$$

where Δ*x* determines the discretization of the space (the numerical solution does not depend on Δ*x*). In the considered case of contact of a rigid indenter with a soft elastic material, effective elastic E^*^ and shear G^*^ modules are defined by the expressions

(6) $$E^{*}=\frac{E}{1-\nu^{2}},\;G^{*}=\frac{2E^{*}(1-\nu )}{2-\nu }.$$

where *E* and *ν*—elastic modulus and Poisson ratio of the material being indented. When profile *g*(*x*) is immersed into an array of non-interacting springs to a depth *d*, compression of an individual spring with coordinate *x* in the normal direction is

(7) $$u_{z}(x)=d-g(x)=d-\frac{x^{2}}{R}.$$

If there is an adhesion in the system, the outer springs are “pulled” to the indenter. As it can be seen from the Figure 7b, these “adhesive” springs reduce the value of the normal force and increase the contact radius *a*, which is calculated according to the Hess rule [31]:

(8) $$\Delta l_{\max}=-\sqrt{\frac{2\pi a\Delta \gamma }{E^{*}}},$$

where Δ*l*_max_ is a magnitude of the enlargement of border springs, and Δ*γ* is an adhesion specific work. As it follows from (7) and (8) with account of Δ*l*_max_ ≡ *u*(*a*)

(9) $$d(a)=\frac{a^{2}}{R}-\sqrt{\frac{2\pi a\Delta \gamma }{E^{*}}}.$$

Normal contact force *F_N_* is defined as a sum of the forces from individual springs in contact (both stretched and compressed):

(10) $$F_{N}=E^{*}\int_{-a}^{a}\;u_{z}(x)dx=2E^{*}\int_{0}^{a}\;\left(d-\frac{x^{2}}{R}\right)dx=\frac{4E^{*}a^{3}}{3R}-\sqrt{8\pi a^{3}E^{*}\Delta \gamma }.$$

The results (9), (10) are exactly the same as the relations from JKR theory [11], concerning the normal contact force *F_N_*, indentation depth *d* and contact radius *a* in the case of adhesive contact. A distinct feature of the experiment, schematically shown in Figure 2, is the indentation at an angle *α* to the elastomer surface where a tangential shift of the indenter is also present together with normal motion. Therefore, after the contact, the springs shown in the Figure 7b undergo a tangential shift. The experiment shows that complex processes of contact boundary restructuring, propagation of elastic waves, etc., occur during the tangential shift. Moreover, the contact becomes asymmetrical (see Videos S2–S4). As we have already noted above, various descriptions of these processes exist in the literature; however, there is still no complete understanding of what happens in adhesive contact during tangential shift. Here, we propose a simple model that neglects the effects of elastic wave propagation and the jump-like reconstruction of the contact boundary, but at the same time allows us to describe the evolution of such quantities as both components of the contact force and the contact area during the process of indentation at an angle precisely enough.

As it follows from numerous experiments [37,38], during tangential shear in the stationary sliding mode, shear stresses preserve their values at almost constant level *τ*_0_ = *F_x_*/*A* and do not depend on indentation depth *d*. The model will be developed from this assumption. The following assumptions should be considered not as a rigorous MDR model but as an estimation of the sliding conditions. Let us consider the stationary sliding mode, with corresponding experimental data shown in Figure 4. Estimation for the average tangential stress can be made using the parameters of the MDR model as

(11) $$\tau =\frac{F_{x}}{A}=\frac{\Delta k_{x}\sum_{cont}u_{x}(x_{i})}{\pi a^{2}},$$

where tangential shift of all springs *u_x_*(*x_i_*) in contact is accumulated in sum. Here, *a* is a radius of a contact, which within a discrete model is defined as *a* = (*n*/2)Δ*x*, where *n* is the total number of springs in contact, and Δ*x* is the space discretization step defined above, which can also be interpreted as a width of the spring or the distance between them. In the case of stationary sliding, when all springs in contact have the same shift *u_x_*(*x*) ≡ $u_{x}^{crit}$, with respect to (5) and (6), we have

(12) $$\tau_{0}=\frac{G^{*}\Delta x\cdot n\cdot u_{x}^{crit}}{\pi n^{2}\Delta x^{2}/4}=\frac{2G^{*}u_{x}^{crit}}{\pi a}.$$

Expression (12) sets the maximum tension of the springs during tangential shear in the form

(13) $$u_{x}^{crit}(a)=\frac{\pi a\tau_{0}}{2G^{*}}.$$

When exceeding this value, springs begin to slide. At this point, we would like to stress again that this is a condition of an “adhesive-like” sliding criterion, which is not valid for the considered system, but will provide a correct estimation of the critical tangential stress. It is worth noting that $u_{x}^{crit}$ depends on the contact radius *a*. Hence, with an increase in the indentation depth, the maximum tangential tension of the springs also increases.

Let us consider the simulation procedure using an example according to scenario (A) that is shown in Figure 2. At a given angle *α* the indenter moves in the normal and tangential directions with velocities *v_z_* = *v*sin*α* and *v_x_* = *v*cos*α*, which specify its normal and tangential displacements as functions of time *t*:

(14) d=vtsinα, x=vtcosα.

During the indenter motion, profile *g*(*x*) is immersed at depth *d*. At the same time, at every iteration, according to Hess rule (8) the springs that are in contact are determined, which defines the contact radius *a* = (*n*/2)Δ*x*. According to (7), the strain of the individual spring in normal direction can be found. Shifts of the springs in tangential direction *u_x_*(*x_i_*) are the same as the shift of the indenter after the moments of the contact of these springs with an indenter, while *u_x_*(*x_i_*) < $u_{x}^{crit}$(*a*).

For those springs where tension *u_x_*(*x_i_*) exceeds critical value $u_{x}^{crit}$(*a*), the tension magnitude is set to be equal *u_x_*(*x_i_*) = $u_{x}^{crit}$(*a*). Normal *F_N_* and tangential *F_x_* forces are calculated as a sum of all forces in contact:

(15) $$F_{N}=E^{*}\Delta x\sum_{cont}u_{z}(x_{i}),\;F_{x}=G^{*}\Delta x\sum_{cont}u_{x}(x_{i}).$$

After the indenter reaches the maximum indentation depth *d*_max_, it is pulled out of the elastomer in the vertical direction with the velocity components *v_z_* = −*v*, *v_x_* = 0. In this case, all the above described actions are performed. Another feature of the simulation is that when new springs come into contact (in the indentation phase), the value of the specific work of adhesion Δ*γ*_0_ is assigned to them, while during the detachment (pull-off phase), a different value Δ*γ*_1_ is used. When the condition Δ*γ*_1_ > Δ*γ*_0_ is true, the developed model reproduces experimentally observed secondary adhesive hysteresis [34,39].

Since in the pull-off phase the value Δ*γ*_1_ is applied only to those springs that have already come into contact, when changing the direction of movement of the indenter, the size of a contact area *A* is preserved for some time, which is clearly observable in experiments on normal indentation (see Figure 3).

The model reported in this subsection, describes the contact between a rigid indenter and an elastic half-space, where the tangential and normal contacts are considered independently of each other. In fact, MDR makes it possible to take into account the thickness of the elastomer being indented [47], as well as the relation between normal and tangential contact, as it was carried out, for example, in [48]. A simpler model was deliberately chosen by us in order to show the main patterns of indentation at an angle, which are described in the next subsection of the work. Description of more subtle effects, such as the effect of tangential shear on contact strength in the normal direction, propagation of elastic waves, friction at the contact boundary, loss of contact area symmetry, etc., requires the construction of more complex models and is not the aim of this work. Some of the abovementioned effects were described in the framework of a dynamic model, proposed by us in [38].

### 4.2. Results of the Simulation

**Figure 8:**
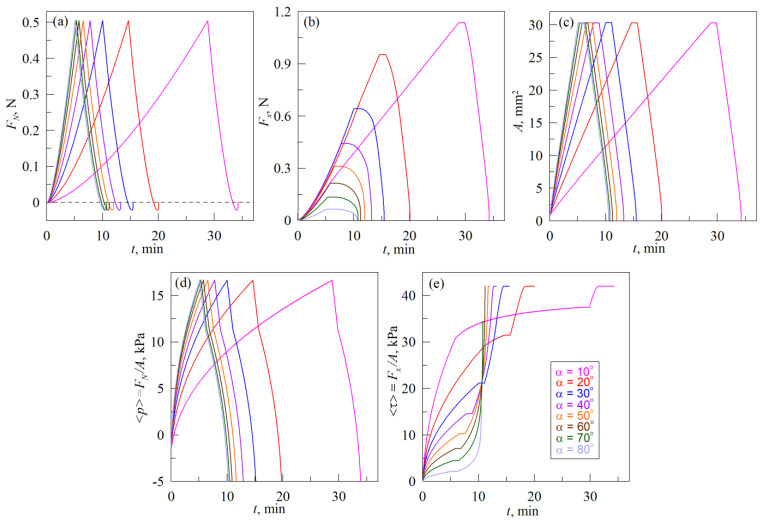
Time dependencies of the normal *F_N_* (**a**) and tangential *F_x_* (**b**) forces, contact areas *A* (**c**), average normal pressure <*p*> (**d**) and average tangential stresses <*τ*> (**e**), obtained in MDR simulations for indentation according to scenario (A), that is explained in Figure 2. Dependencies corresponding to the experiment with the same angle *α* (from 10° to 80°), plotted in different panels of the figure in the same color. Parameters of simulations: elastic modulus of the half-space *E* = 0.324 MPa, its Poisson ratio *ν* = 0.48, specific adhesion work during indentation Δ*γ*_0_ = 0.01 J/m^2^, and during pull-off Δ*γ*_1_ = 0.15 J/m^2^, stationary tangential stresses in the sliding mode *τ*_0_ = 42 kPa, space discretization parameter Δ*x* = 10^−6^ m. All other parameters such as indenter radius *R*, its velocity of movement *v*, maximal indentation depth *d*_max_, as well as indentation angle *α* are exactly the same as in the experiment.

Figure 8 shows the dependencies of the main parameters, obtained from the simulation of indentation according to scenario (A), the schematics of which are shown in Figure 2. The dependencies, shown in Figure 8, relate to the conditions of a real-life experiment with the experimental data presented in Figure 5.

Figure 8 does not show the case with pure normal indentation at *α* = 90°. Additionally, the panel (f) that shows relation *L_vertical_*/*L_horisontal_* in experimental Figure 5 is also absent in the Figure 8 due to the fact that in MDR simulations the contact area is considered as circular and ratio *L_vertical_*/*L_horisontal_* is always equal to one.

As it follows from the Figure 8, the simulation results reproduce performed experiment qualitatively correct (see Figure 5). The most important differences between the experiment and simulation, as well as possible explanations for such differences and suggestions for further improvement of the model, are described below.

(1) As Figure 5a shows, the adhesive strength of the contact in the normal direction decreases with decreasing of the angle value *α*. Here, by adhesive strength we mean the absolute value of the normal force |*F_N_*_,min_| during pull-off phase at *F_N_* < 0 N, i.e., the force caused by adhesion, because the same external force must be applied to completely destroy the contact. The maximum adhesive strength is observed in a purely normal contact (see dependence *F_N_*(*d*) in Figure 3a for pull-off phase). At the presence of tangential shift, the strength of the contact in the normal direction decreases. In the simulation, we considered tangential and normal contact independently, therefore, for all angles *α* magnitudes of |*F_N,_*_min_| are the same (see Figure 8a). Moreover, independent consideration of normal and tangential contact leads to the situation where dependencies *A*(*t*) in Figure 8c at the beginning of the pull-off phase exhibit intervals with the constant size of the contact area *A*. Tangential force *F_x_* also does not change within these time intervals of constant contact area *A*, due to the absence of the tangential movement during the indenter pull-off in the pure normal direction. This constrain can be avoided by introducing the coupling between normal and tangential contact as it was carried out in [48] for instance. However, application of the criterium, proposed in [48], in our simulation did not lead to the expected results and therefore additional studies on this matter are needed.

(2) Maximum values of the normal *F_N_* and tangential *F_x_* forces, as well as the size of a contact area *A* and average contact pressure <*p*> obtained in experiments exceed those from simulations. The reason for this is that in the experiment, indentation was performed in an elastomer layer with a limited thickness *h* = 5 mm, while simulation was carried out for a half-space, for which *h* → ∞. In the case of an elastic layer, the stiffness of the contact is significantly higher (especially for elastomers) [32], which leads to increased values compared to the half-space case. To take into account the limited thickness of the layer, it is possible to use the generalized MDR proposed in a recent work [47], which, however, contains a description of the modeling procedure only for normal contact.

(3) In the pull-off phase of the detachment of the indenter from the elastomer in the normal direction the tangential stresses in all cases increase to the maximum stationary value *τ*_0_ = 42 kPa (see Figure 8e) in the simulation, while in the experiment stresses <*τ*> in the pull-off phase are characterized by a rapid growth (see Figure 5e). In simulations, the limit for *τ*_0_ values caused by the use of the springs sliding criteria (12), (13)—during the pull-off phase contact radius *a* decreases, therefore, according to (13), there comes a moment when all the springs in contact begin to slide due to a decrease in the value of their critical tension $u_{x}^{crit}$, and thus providing constant tangential stresses *τ*_0_ over all contact area. The rapid growth of stresses *τ*_0_, observed in experiments during pull-off, may be related to contact strengthening in time, and also to the viscoelasticity of the elastomer. Strengthening of the contact leads to the fact that the value *τ*_0_ increases as well as adhesive strength in normal direction |*F_N_*_,min_|. At the same time, viscoelasticity leads to a decrease in the velocity of rubber slip over the indenter, and, as a result, to the growth of *τ*_0_ during the indenter motion. In the used estimation based on MDR, it is possible to take into account both contact strengthening (by increasing the value of the specific work of adhesion Δ*γ* with time) and viscoelasticity (by using Kelvin–Voigt elements instead of springs shown in Figure 7b). However, before such a modification of the model, it is necessary to find out the true causes of the described behavior first, which requires additional experiments that are beyond the scope of the proposed work.

(4) The experiment shows the moments of abrupt increase in the contact area *A* when new regions of rubber are attached to the indenter, and the rearrangement of the contact boundary develops in different ways on the front and back sides of the contact. It is clearly observable in the experimental dependencies *A*(*t*), <*p*>(*t*) and *τ*(*t*) presented in the Figure 5 for an angle *α* = 10° (see also Supplementary Video S3), at conditions close to tangential shear, as well as during pure tangential contact (see Figure 4 and Supplementary Video S2). In the simulation, the contact area grows smoothly, since its increase occurs only due to indentation in the normal direction (adhesive JKR contact). This simplification of the model is related to the paragraph (2) above, when the normal and tangential contact are considered independently.

Note that mentioned simplifications of the model lead to visible differences between experiment and simulation only when the angle at which the indentation is performed deviates significantly from the value *α* = 90°, that is corresponding to normal contact. In the case of indentation at angles close to 90° (for example, 80°, 70° and 60°), the experiment and theory give almost qualitatively identical results, since at such angles, in the indentation phase newly attached rubber regions move together with the indenter without slipping.

Taking into account the spring slip criterion (13), and assuming that indenter trajectory is known (14), the conditions when slippage of the springs begins can be easily determined. Now, we consider indentation according to scenario (A) in the phase of indentation, when the specific work of adhesion Δ*γ*_0_ is small, therefore, with sufficient accuracy, the contact can be considered as non-adhesive in the normal direction. At this, the contact radius is defined as [49]

(16) $$a=\sqrt{Rd}.$$

**Figure 9:**
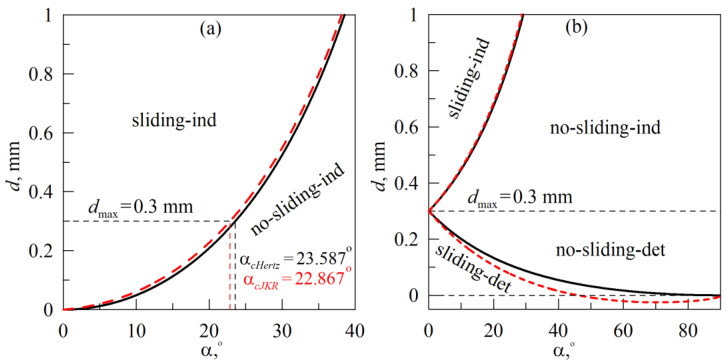
Dependences of the critical indentation depth *d*_crit_, at which slip begins, on the indentation angle *α*. Panel (**a**) shows dependencies for indentation scenario (A), panel (**b**) for scenario (B), both indentation scenarios are shown schematically in Figure 2.

According to (13) and (14), sliding of springs in contact in the phase of immersion of the indenter at an angle *α* occurs when the general displacement of the indenter *x* = *vt*cos*α* becomes equal to critical displacement $u_{x}^{crit}$(*a*) (13). If the contact radius is defined according to (16), it can be determined that slippage will start if the indentation depth *d* exceeds a critical value:

(17) $$d_{\mathrm{crit}}=\frac{\pi^{2}\tau_{0}^{2}R}{4{\left(G^{*}\right)}^{2}}\tan^{2}\alpha ,$$

which depends on the indentation angle *α*. According to (17), at indentation angles *α* < 90° slipping will occur anyways (with the growth of *α* slipping appears at larger indentation depth *d*). Dependence *d*_crit_(*α*) is shown in Figure 9a in solid line.

Figure 9a represents a diagram with two regions, the “sliding-ind” (here ”ind” denotes indentation) region, characterized by sliding of the springs, and the “no-sliding-ind” region, where during the entire indentation phase, new springs move in the tangential direction together with the indenter without slipping after attachment. The solid curve in the figure shows the dependence *d*_crit_(*α*), defined by the expression (17). According to this dependence, at maximal indentation depth *d*_max_ = 0.3 mm sliding in the indentation phase takes place if *α* < *α_cHertz_* ≈ 23.59°. However, main disadvantage of estimation (17) is the definition of the contact radius *a* (16) obtained for non-adhesive contact. At small magnitudes of the specific work of adhesion Δ*γ*, such an approach is precise enough. However, if Δ*γ* is not necessarily small, for example in the pull-off phase, adhesion plays a crucial role. When the simple definition (16) is not adopted, dependence *d*_crit_(*α*) is defined by the solution of the system of equations:

(18) $$\left\{ \begin{array}{l} d_{\mathrm{crit}}=\frac{\pi a\tau_{0}\tan\alpha }{2G^{*}},\\ d_{\mathrm{crit}}=\frac{a^{2}}{R}-\sqrt{\frac{2\pi a\Delta \gamma }{E^{*}}}, \end{array}\right.$$

where first equation follows from (13) and (14) at *x* = $u_{x}^{crit}$(*a*), while second equation is a relation (9), needed for contact radius *a* determination. Obtained from (18) dependence *d*_crit_(*α*), calculated at Δ*γ* = 0.01 J/m^2^ for the indentation phase (see parameters in the caption of Figure 8), is shown in Figure 9a in dashed line and located slightly above the “non-adhesive” dependence (17). Thus, taking into account adhesion widens the diagram region related to the absence of sliding (“no-sliding-ind”), at the same time at the maximum indentation depth *d*_max_ = 0.3 mm slips occur at smaller, comparing to non-adhesive case, angle *α_cJKR_* ≈ 22.87°.

Dependencies shown in Figure 8, automatically relate to the above analysis of the slippage criterion, since they are obtained from the simulations. However, experimental data presented in Figure 5 also show the absence of the slipping mode at large angles *α*, that confirms Equations (17) and (18). Note that the above-mentioned complex dynamic processes take place within the slip mode, associated with a jump-like increase in the contact area, the propagation of elastic waves, etc. Qualitatively, these processes were described in our recent paper [38], where the influence of the indentation depth *d*_max_ on the tangential contact in the presence of adhesion was studied. In [38], an experiment was also conducted and a numerical model was proposed.

**Figure 10:**
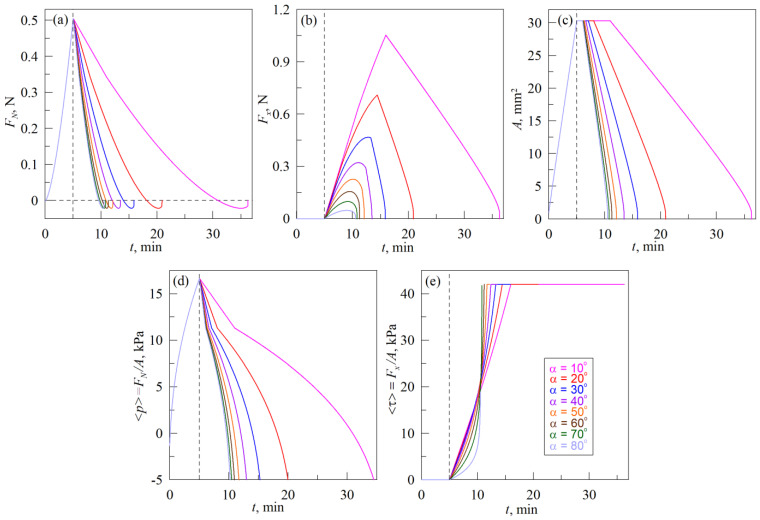
Time dependencies of the normal *F_N_* (**a**) tangential *F_x_* (**b**) forces, contact areas *A* (**c**), average normal pressure <*p*> (**d**) and average tangential stresses <*τ*> (**e**), obtained from the MDR simulations for the scenario (B), schematics of which are shown in Figure 2. Dependencies corresponding to the experiment with the same angle *α* (from 10° to 80°), plotted in different panels of the figure in the same color.

Figure 10 shows the results of the simulation according to scenario (B), shown in Figure 2. Thus, results presented in Figure 10, should be compared to the experimental data shown in Figure 6. As it follows from the comparison, the simulation results and experimental data differ similarly to the scenario (A), therefore we will not discuss them in detail again. Let us discuss, however, the sliding criterion, similar to the above described expressions (17) and (18), that were obtained for scenario (A). In scenario (B) after the indentation to the maximal depth *d*_max_ = 0.3 mm, the indenter is shifted along the trajectory that is defined by equations:

(19) $$d=d_{\max}-v\tilde{t}\sin\alpha ,\;x=v\tilde{t}\cos\alpha ,$$

where $\tilde{t}$ is time, measured from the moment when the indenter stopped to immerse, i.e., from the beginning of the tangential shift. Expressions (13), (16) and (19) together lead to the quadratic equation for critical value of the indentation depth *d*_crit_, at which springs start to slip:

(20) $$d_{\mathrm{crit}}^{2}-\left[2d_{\max}+R{\left(\frac{\pi \tau_{0}\tan\alpha }{2G^{*}}\right)}^{2}\right]d_{\mathrm{crit}}+d_{\max}^{2}=0$$

with two positive roots.

The smaller root *d*_crit_ ≤ *d*_max_ defines the slip condition when pulling-off the indenter according to the scenario (B). In Figure 9b, this root relates to the lowest solid line *d*_crit_(*α*), that divides the diagram region *d* < *d*_max_ = 0.3 mm into two parts: “sliding-det” with sliding (here “det” denotes detachment) and “no-sliding-det” without sliding. The diagram region at *d* < *d*_max_ relates to the performed experiment (see Figure 6) and simulation (see Figure 10). As it follows from Figure 9b, during the indenter pull-off from the initial indentation depth *d*_max_ at an angle *α,* if *α* < 90° springs begin to slip in a certain moment of time.

Moreover, the smaller the angle, the earlier the slip begins, since the depth of indentation *d* in the experiment decreases, starting from the value of *d*_max_ (see the first equation in (19)). Note that with a purely tangential movement (*α* = 0°), slippage on the diagram occurs already at *d* = *d*_max_, since the indentation depth *d* in the experiment is equal to the maximum value *d*_max_ and does not change (case with the absence of normal movement of the indenter, is shown in Figure 4). When the indenter is pulled-off in the normal direction (*α* = 90°), there is no slippage (case with the absence of tangential movement, is shown in Figure 3). The above conclusions are confirmed by the experiment, in which a region *τ*_0_ = const is observed within whole range of angles *α* (see Figure 6e). The length of this region, however, significantly decreases with the growth of an angle *α*. Similar behavior is also observed in simulations (see Figure 10e).

Dependence *d*_crit_(*α*), which is located above the value *d* = *d*_max_ = 0.3 mm in the Figure 9b, relates to the situation when the indenter is not withdrawing from the elastomer after reaching the critical indentation depth, but continues to immerse into elastomer, moving at an angle *α* instead of indentation in the normal direction. This curve also divides the area of the diagram at *d* > *d*_max_ = 0.3 mm into two parts with (”sliding-ind”) and without (“no-sliding-ind”) sliding. Plotted together by solid lines in the Figure 9b, both dependencies *d*_crit_(*α*) define a complete diagram of indentation modes, for the cases of indentation and pull-off of the indenter at an angle *α* from the initial value *d* = *d*_max_ = 0.3 mm.

The refined dependence *d*_crit_(*α*) with taking into account the adhesion in the normal direction is given by the solution of the system of equations (compare with (18))

(21) $$\left\{ \begin{array}{l} d_{\mathrm{crit}}=d_{\max}\pm \;\;\frac{\pi a\tau_{0}\tan\alpha }{2G^{*}},\\ d_{\mathrm{crit}}=\frac{a^{2}}{R}-\sqrt{\frac{2\pi a\Delta \gamma }{E^{*}}}, \end{array}\right.$$

where in first equation “–” sign relates to the scenario (B), when the trajectory of the indenter is defined by Equation (19). The choice of the “+” sign in this equation describes a situation in which the indenter after reaching the maximum indentation depth *d*_max_ during the indentation in the normal direction continues immersing into the elastomer at an angle *α*. Dependencies, defined by Equation (21), are shown in the Figure 9b by dashed lines. The upper dashed curve was obtained at a smaller value of the specific work of adhesion Δ*γ* = Δ*γ*_0_ = 0.01 J/m^2^, because it relates to the indentation phase. Therefore, the difference between the “adhesion-free” curve (solid line) and the curve with adhesion in the normal direction (dashed line) is not significant here. However, for the pull-off phase (curves below *d*_max_ value), the difference is more significant, since the pull-off phase is characterized by a significantly larger value Δ*γ* = Δ*γ*_1_ = 0.15 J/m^2^. Moreover, the dashed curve, obtained by taking into account the adhesion in the normal direction, in a certain range of angles *α* located below the *d* = 0 mm axis, since due to adhesion contact it also exists at *d* < 0 mm.

Besides the experiments described above performed with an indenter with a radius of *R* = 30 mm, we conducted a similar series of experiments with an indenter of a larger radius *R* = 100 mm. In order to not overload the article, we do not describe these experiments here, but they are available as video files in the Supplementary Materials (Videos S5–S8), which show the evolution of the contact area and main parameters. The difference between the corresponding experiments with indenters of different radii is only the value of the radius *R*, all other conditions of the experiment were the same. In addition to the video files, the Supplementary Materials contain a file named “Figures S1–S4” with the dependences of the main parameters of the system obtained for the indenter with the radius *R* = 100 mm. Presented dependencies are similar to the data described above for the case *R* = 30 mm.

## 5. Conclusions

We performed two series of experiments, in which a steel indenter is indented into a soft elastomer at an angle *α* to its surface. In the first series of experiments, the indentation is carried out at an angle, i.e., the indenter immerses into the elastomer, and the contact area increases. The second series relate to the pull-off phase, when the indenter that is immersed to a certain depth is pulled out of the elastomer when moving at an angle the contact area thereby decreases. With the aim to obtain the full picture, experiments were separately conducted for cases with normal (*α* = 90°) and tangential (*α* = 0°) contact. Experiments in which the indenter is immersed at an angle are of particular interest, since in this case, during the entire indentation, new areas of the substrate that are not loaded in the tangential direction are involving in the contact, which leads to an inhomogeneous distribution of tangential stresses in the contact zone. The case where the indenter is pulled-off at an angle is a simpler situation, since the movement occurs from a fixed depth of the elastomer, while the entire contact is loaded during tangential movement.

For both cases, a simple numerical model based on the method of dimensionality reduction (MDR) was developed. The model makes it possible to obtain the dependences of the main parameters that are also were measured in the experiment (both components of the contact force and the contact area) on the angle *α* at which indentation was performed and on the displacement of the indenter in the tangential and normal directions. Although the model is simple, it produces correct dependencies that are also in good agreement with the experimental results. The simulation allowed us to build an indentation regime diagram, which contains areas with the presence and absence of slippage. In the presence of slip, complex dynamic effects such as the propagation of elastic waves and the constant rearrangement of the contact boundary are observed. The description of such effects is impossible within the framework of the proposed quasi-static contact model. Due to the complexity of these processes, additional research is needed, which is, however, beyond the scope of the presented study. In the no-slip (stick) mode, the simulation shows a much better agreement with the experiment, since the dynamic effects mentioned above are absent.

The performed study may help to better understand what happens when a contact occurs and when it is destroyed if the contacting objects move at an angle to one another. Such a situation is typical for biological organisms that use adhesion to move along inclined surfaces. An important feature of our work is that we separately consider the cases of contact occurrence (when indenting into an elastomer) and its destruction (pulling an indenter out of an elastomer), as these two phases are necessary for animals to complete their movement. Even though animals were not used in the presented study, the considered system of contact of a rigid object (indenter) with a much softer elastomer is very close to the contact of the adhesive surfaces of animals (for example, the paws of some frog species) and hard surfaces along which they successfully move (stone, wood, etc.). In this case, the soft material, due to its good ability to deform, is able to fill up existing roughness in the solid body during contact even with the application of small forces. Due to such filling, there is a significant increase in the real contact area and, as a consequence, the adhesive strength at detachment. These features allow animals to form a strong adhesive contact that can hold their weight on inclined and even vertical surfaces.

## Data Availability

The datasets generated for this study are available upon request from the corresponding authors.

## Supplementary Materials

The following supporting information can be downloaded at: https://www.mdpi.com/article/10.3390/biomimetics8060477/s1, Video S1: An experiment concerning indentation in the normal direction of a steel indenter with a radius *R* = 30 mm into a layer of TARNAC CRG N3005 rubber with a thickness *h* = 5 mm. The video shows the evolution of the contact zone, as well as the dependence of the normal force *F_N_* and the contact area *A* on the indentation depth *d*, where the regions of the dependences corresponding to the indentation and the pull-off phase are shown in red and blue, respectively. The velocity of the indenter movement in the indentation and pull-off phases *v* = 1 μm/s. The upper left corner shows the real time *t* from the beginning of the experiment in seconds (from the moment of the first contact between the elastomer and the indenter). Video relates to Figure 3 in the article. Video S2: An experiment: concerning tangential shift of the indenter. Initially steel indenter with *R* = 30 mm immersed into a layer of TARNAC CRG N3005 rubber with a thickness h = 5 mm to the depth *d*_max_ = 0.3 mm. Then, the indenter is shifted to a distance *x* = 3 mm in the tangential direction at a fixed indentation depth *d*_max_, after which it is pulled out until the complete detachment of the contact in the normal direction. Indenter’s velocity in all phases of the experiment *v* = 1 μm/s. The video shows the evolution of the contact zone, as well as the time dependencies of the normal force *F_N_*, tangential force *F_x_* and contact area *A*. The top left corner shows the current indentation depth *d* and tangential shift *x*. Video relates to Figure 4 in the article. Video S3: An experiment performed according to the scenario (A), schematic of which is shown in Figure 2 in the article. Indenter with the radius *R* = 30 mm was immersed to depth *d*_max_ = 0.3 mm into a layer of TARNAC CRG N3005 rubber with a thickness *h* = 5 mm. In the indentation phase, the indenter was shifted at an angle *α* to the elastomer surface with the velocities *v_z_* in normal and *v_x_* in tangential direction, where *v_x_* = *v*cos*α*, *v_z_* = *v*sin*α*, and $v=\sqrt{v_{z}^{2}+v_{x}^{2}}=1$ μm/s. The video shows the series of experiments with angles *α* = 80°, 70°, 60°, 50°, 40°, 30°, 20° and 10°. After the indentation to the depth *d*_max_ indenter was pulled off the elastomer in the normal direction with the velocity *v* = 1 μm/s. Separate panels in video show time dependencies of the normal *F_N_* and tangential *F_x_* forces, contact area *A*, average contact pressure <*p*> = *F_N_*/*A* and average tangential stresses <*τ*> = *F_x_*/*A*. In addition, the lower left panel shows the evolution of the contact zone, it also shows the current values of the indentation depth *d*, the tangential shift of the indenter *x*, and the time *t* that has passed since the beginning of indentation (since the first contact between the rubber and the indenter). Video relates to Figure 5 in the article. Video S4: An experiment performed according to the scenario (B), schematics of which is shown in Figure 2 in the article. First, indenter with the radius *R* = 30 mm was immersed to the depth *d*_max_ = 0.3 mm into a layer of TARNAC CRG N3005 rubber with a thickness *h* = 5 mm at motion in normal direction with the velocity *v* = 1 μm/s. After this indenter was pulled off from the elastomer at an angle *α* to its surface with the velocities *v_z_* in normal and *v_x_* in tangential directions, where *v_x_* = *v*cos*α*, *v_z_* = – *v*sin*α*, and $v=\sqrt{v_{z}^{2}+v_{x}^{2}}=1$ μm/s. The video shows the series of experiments with angles *α* = 80°, 70°, 60°, 50°, 40°, 30°, 20° and 10°. Separate panels in the video show time dependencies of the normal *F_N_* and tangential *F_x_* forces, contact area *A*, average contact pressure <*p*> = *F_N_*/*A* and average tangential stresses <*τ*> = *F_x_*/*A*. In addition, the lower left panel shows the evolution of the contact area, and it also shows the current values of the indentation depth *d*, the tangential shift of the indenter *x*, and the time *t* that has passed since the beginning of indentation (since the first contact between the rubber and the indenter). Video relates to Figure 6 in the article. Video S5: Same as the Video S1, but for the indenter with radius *R* = 100 mm. Video relates to Figure S1 in Supplementary Materials. Video S6: Same as the Video S2, but for the indenter with radius *R* = 100 mm. Video relates to Figure S2 in Supplementary Materials. Video S7: Same as the Video S3, but for the indenter with radius *R* = 100 mm. Video relates to Figure S3 in Supplementary Materials. Video S8: Same as the Video S4, but for the indenter with radius *R* = 100 mm. Video relates to Figure S4 in Supplementary Materials. Figures S1–S4: Experimental dependencies of the main parameters obtained for the indenter with the radius *R* = 100 mm. Figures S1–S4 similar to Figure 3, Figure 4, Figure 5 and Figure 6 presented in the article (In the article these dependencies are obtained for the indenter with the radius of *R* = 30 mm).
